# Controlled-release nitrogen combined with ordinary nitrogen fertilizer improved nitrogen uptake and productivity of winter wheat

**DOI:** 10.3389/fpls.2024.1504083

**Published:** 2025-01-07

**Authors:** Muhammad Fraz Ali, Ruifeng Han, Xiang Lin, Dong Wang

**Affiliations:** State Key Laboratory for Crop Stress Resistance and High-Efficiency Production, College of Agronomy, Northwest A & F University, Yangling, Shaanxi, China

**Keywords:** controlled released nitrogen fertilizer, grain yield, nitrogen accumulation, soil nitrogen supply, winter wheat

## Abstract

**Background:**

Blending controlled-release nitrogen fertilizer (CRNF) with ordinary nitrogen fertilizer (ONF) is a strategic approach to improve winter wheat nutrient management. This blend provides nitrogen (N) to winter wheat in a balanced and consistent manner, ensuring long-term growth, reducing nutrient loss due to leaching or volatilization, and increasing N use efficiency (NUE).

**Aims:**

CRNF aims to enhance N application suitability, optimizes soil nutrient dynamics, and its widespread use can boost crop NUE and yield. The study investigates how different CRNF and ONF blending ratios affect soil N content, winter wheat growth, and yield.

**Methods:**

The experiment used two N application rates of 192(N_1_) and 240(N_2_) kg ha^-1^ of ONF, with five different blending ratios CRNF. The proportions of CRNF were 0%(F_1_), 30%(F_2_), 50%(F_3_), 70%(F_4_), and 100%(F_5_), respectively. The effects of changes in soil nitrate concentration, dry matter accumulation, N uptake, and transportation at various growth stages and yield were analyzed.

**Results:**

CRNF at the jointing and anthesis stages helps maintain nitrate N levels throughout the growth cycle. Compared to full CRNF application at different N rates, this method also reduces nitrate N leaching in the soil. The 0-60 cm soil layer was primarily influenced by increasing the proportion of CRNF, especially from jointing to maturity. CRNF promotes a higher plant population during the turning green and jointing stages by increasing soil N content, thereby establishing a strong yield foundation for winter wheat. It increases winter wheat N accumulation and correlates positively with soil N content during key growth stages.

**Conclusion:**

Winter wheat grain yield has increased, with significant yield increases observed at 70% blending with a higher amount of N at 240 kg ha^-1^ and achieved a 2.8% increase in NUE and a 3.0%-15.3% increase in grain yield. In order to improved winter wheat yields through effective N utilization, N2 application (240 kg ha^-1^) with the combination of (F_4_) 30% ONF + 70% CRNF would be recommended for northwest region of Shaanxi province in China. By increasing the amount of N accumulation at the anthesis stage, N transport is significantly increased after anthesis, and N accumulation and distribution ratio in grains are significantly increased at maturity.

## Highlights

- Increasing CRNF increased soil nitrate N content and reduced leaching across winter wheat growth.
- Combination of CRNF and ONF increased DM accumulation from jointing to maturity in winter wheat.
- CRNF at 70% combined with ONF increased N accumulation, N uptake and N use efficiency of winter wheat.
- Using 70% CRNF combined with ONF at 240 kg ha^-1^ overcame the bottleneck of increasing the yield and NUE.

## 1 Introduction

Farmers were using fertilizers from the ancient time itself, but the long-term use of fertilizers had affected the soil fertility which led to pollution of water, air, and soil. Nitrogen (N), particularly in the form of urea, is an essential nutrient fertilizer, which has been critical for increasing plant growth and yield and is widely used to maintain soil fertility (Dimkpa et al., 2020). China is the largest user of urea, which has been used to increase crop production and address food security challenges (Snapp et al., 2023; Gu, 2023). According to statistics, synthetic N input to field crops in China has increased by 215% since 1980 (Zhang et al., 2022a). However, crop N uptake has only increased by 109% in the last 30 years, resulting in lower N fertilizer recovery efficiency (Yan et al., 2014). When urea is applied to farmland, nutrients are rapidly released, and a significant portion is lost due to ammonia volatilization and nitrate N leaching, which are the primary causes of low N utilization efficiency (NUE) (Hussain et al., 2022; Xiao et al., 2019). Furthermore, applying more N than the crop requirement may disturb groundwater, surface water, and atmosphere via N leaching, runoff, and volatilization (Ntinyari et al., 2022), resulting in a waste of resources and energy, as well as an increase in environmental pollution and production costs (Hussain et al., 2023b). Additionally, low NUEs lead to lower economic returns for growers from their fertilizer investments (Snapp et al., 2023).

Global reliance on winter wheat as a primary food source is increasing, with demand expected to double in the 21^st^ century, exceeding current production requirements (Wu et al., 2023; Erenstein et al., 2022). Wheat production is reducing due to numerous factors such as environmental stresses, late sowing, lack of high-quality seeds, climate variability, and insect pests. Fertilizer use must be optimized to address environmental concerns while ensuring global food security (Anderson et al., 2020). The Chinese Ministry of Agriculture introduced the ‘Zero Increase Action Plan’ a strategy to boost crop yields and mitigate environmental issues without increasing fertilizer usage (Liu et al., 2016). This policy proposed several management practices for optimal N fertilizer application, one of which is the development of a controlled-release N fertilizer (CRNF), which provides a new theoretical method for simple and efficient fertilization technology for field crops (Vejan et al., 2021).

Controlled-release urea (CRU) is a new type of urea fertilizer with a physical coating that ensures controlled N release into the soil over time to ensure a continuous supply of N, thereby improving crop N demand and supply coordination (Zhang et al., 2022; Vejan et al., 2021). It is frequently used as a basal fertilizer because it saves labor costs for split application compared to regular urea and improves the synchronization of N supply to soil (Geng et al., 2015; Ma et al., 2021). Various studies have confirmed that the application of CRU significantly increased the NUE and yield of winter wheat (Yang et al., 2011; Zhang et al., 2022; Ma et al., 2021; Zheng et al., 2016; Zhu et al., 2020). For example, Zhou et al. (2023) proposed CRU optimization and stated that CRU proportion was more critical than CRU longevity, reporting that under N treatments of 157.5, 180, and 225 kg ha^-1^, the optimal ranges of CRU proportion were 41~64%, 38~64%, and 24~40% for wheat yield, respectively. In another study, Li et al. (2020) reported that irrigation management and CRU significantly impacted maize growth and development, enhancing plant dry matter (DM) accumulation and N uptake. Similarly, Zheng et al. (2016) reported in a 5-year study of CRU in a wheat-maize cropping system that the application of CRU as a basal fertilizer outperformed normal urea (NU) applied as split fertilization with the same quantity of N application.

Winter wheat has a more extended growing season and requires more labor, which is becoming expensive, so it is vital to promote the use of CRU in wheat cultivation and save labor costs. The CRU application is sub-optimal, so the combination of CRU and NU can make soil N supply more consistent with winter wheat N demand, increase N absorption, and affect wheat grain quality characteristics (Zhang et al., 2018; Zhou et al., 2019). A combination of CRU and NU should be used as a base fertilizer for winter wheat for winter wheat balances N supply, overcoming initial slow release and ensuring long-term availability (Zhang et al., 2022; Li et al., 2018). As a result, this combination is a potential N fertilizer management strategy for increasing wheat grain yield and protein content, and its mechanism should be thoroughly investigated.

Previous research has primarily focused on the release timing of CRNF related to crop N uptake and the resulting yield benefits. However, more research is needed to understand how CRNF responds to variations in soil N availability and N supply capacity. We hypothesized that combining CRNF and ONF would make the soil N supply more consistent with the N demand of winter wheat, increase N absorption in winter wheat, and possibly affect grain yield. Based on this, the current experiment used different blending ratios of CRNF and ONF to explore the soil N supply situation during the entire growth period of winter wheat. The current study aims to investigate winter wheat growth under different soil N supply conditions and to analyze how different blending ratios of CRNF and ONF can affect soil nutrient dynamics and winter wheat productivity. The regulatory pathways and mechanisms of different CRNF and ONF mixing ratios on N accumulation and transport, and the physical properties of winter wheat will be investigated, in order to develop high-quality, appropriate, and effective fertilization solutions.

## 2 Materials and methods

### 2.1 Experimental site and materials

**Figure 1:**
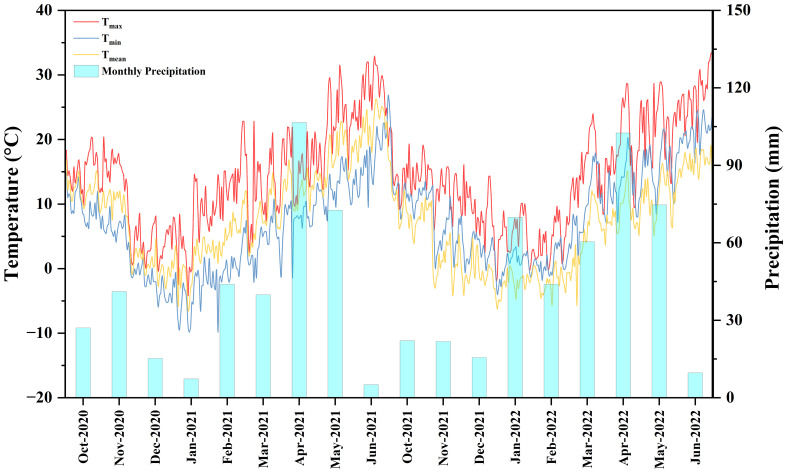
Daily precipitation and temperature during the winter wheat growing season from 2020-2022.

The experiment was conducted during two wheat growing seasons from 2020 to 2022 at the an experimental demonstration site of Northwest A&F University, in Liangma Village, Wugong County, Xianyang City, Shaanxi Province (34°21’N, 108°02’E). The experimental site has an altitude of 443 m, with an average annual rainfall of 504.6 mm and a temperature of 14.37°C. The cropping system of the experimental site was summer maize-winter wheat rotation. All maize residue was crushed during the experiment and returned to the field after harvest. The average daily temperature and rainfall in the two-winter wheat cropping season (2020-2022) are shown in **Figure 1**. The physical and chemical properties of the soil (0-20 cm) in the soil layer before sowing for the two years are shown in **Table 1**.

**Table 1:** Soil physical and chemical properties of 0-20 cm soil layer before sowing.

Year	Organic matter (g kg^-1^)	Total nitrogen (g kg^-1^)	Hydrolysable nitrogen (mg kg^-1^)	Available phosphorus (mg kg^-1^)	Available potassium (mg kg^-1^)
2020-2021	1.5	0.97	64.1	10.4	220.2
2021-2022	1.6	0.93	54.5	20.7	231.5

This study used the winter wheat cultivar ‘Xinong 20’, which is widely grown locally. The N fertilizers included in this study were resin-coated urea (CRNF 44%, N) and ordinary urea (ONF 46.4% N). The controlled-release period was 90 days, and it was supplied by Weituoer Company, Yangling District, Shaanxi Province. Potassium chloride (K_2_O 60%) and heavy superphosphate fertilizer (P_2_O_5_ 46%) were utilized as potassium and phosphate fertilizer, respectively.

### 2.2 Experimental design

The experiment used a randomized complete block design with two factors and three replications. The factors include two N application rates (N_1_, 192 kg ha^-1^; N_2_ 240 kg ha^-1^) of ONF and different blending ratios of CRNF and ONF are set under two N application rates, which were 100% ONF (F_1_), 70% ONF + 30% CRNF (F_2_), and 50% ONF + 50% CRNF (F_3_), 30% ONF + 70% CRNF (F_4_), and 100% CRNF (F_5_). The N_2_ (240 kg ha^-1^) is the local conventional N application amount while N_1_ (192 kg ha^-1^) is a 20% reduction in N. The application rate of phosphorus and potassium fertilizers in all treatments was the same, 120 kg ha^-1^. Nitrogen, phosphorus, and potassium fertilizers were applied as basal fertilizers in every treatment. The area of the test plots was 10 m length × 2 m width (20 m^2^). The winter wheat for 2020-2021 was planted on October 26 and harvested on June 11, while the winter wheat for 2021-2022 was planted on October 17 and harvested on June 9. Irrigation, weeding, and other management practices during the growth period of winter wheat are the same as those of local farmers.

### 2.3 Sampling and measurements

#### 2.3.1 Soil nitrogen dynamics

Two core soil samples were collected from the 0-10, 10-20, 20-30 and 30-40  cm soil layers using an auger having an inner diameter of 4.0 cm. Soil samples were collected and stored in a labeled plastic bag from each plot during the overwintering, greening, jointing, and anthesis stages of winter wheat. Soil samples were collected from the 0-200 cm soil layer at maturity stage. After air drying, the soil sample was sieved and weighed 5.0 g (accurate to 0.001), were extracted with 50 mL of 1 mol L^-1^ KCl, shaken for 30 minutes, then filtered, and the soil nitrate N and ammonium N content were measured using the continuous flow analyzer (Model AA3-A001-02E, Bran-Luebbe, Germany) in the laboratory (Markus et al., 1985).

#### 2.3.2 Dry matter accumulation and distribution

Plant samples were taken during the overwintering, greening, jointing, anthesis, and maturity stages to determine the aboveground DM accumulation and distribution. Stem, leaves, and spike were sampled at overwintering, greening, jointing, and anthesis stages, while stem, leaves, rachis+glume, and grain were sampled at the maturity stage. The separated plants were placed in an oven at 105°C for 30 minutes and dried at 70°C to a constant weight. The samples were then weighed separately. The calculation formulas for each indicator are as follows:


(1) $$\begin{array}{c} DM\;distribution\;to\;grains\;at\;maturity\;stage\left(\%\right)\\ =\frac{Distribution\;of\;dry\;matter\;in\;grains\;at\;maturity\;stage}{Accumulation\;above\;ground\;dry\;matter\;at\;maturity\;stage}\times 100 \end{array}$$

(2) $$\begin{array}{c} Contribution\;rate\;of\;assimilates\;stored\;in\;vegetative\;organs\;before\;anthesis\;to\;grain(\%)\\ =\frac{assimilates\;transported\;in\;vegetative\;organs\;stored\;before\;anthesis}{dry\;weight\;of\;grains\;at\;maturity}\times 100 \end{array}$$

(3) $$\begin{array}{c} Contribution\;rate\;of\;assimilates\;to\;grains\;after\;anthesis\;\left(\%\right)=\\ \frac{allocation\;amount\;of\;assimilates\;in\;grains\;after\;anthesis}{dry\;weight\;of\;grains\;at\;maturity}\times 100 \end{array}$$

(4) $$\begin{array}{c} Storage\;and\;assimilation\;of\;vegetative\;organs\;before\;anthesis\left(kg\;ha^{-1}\right)\\ =dry\;matter\;at\;anthesis\;-dry\;matter\;at\;maturity \end{array}$$

(5) $$\begin{array}{c} Distribution\;of\;assimilates\;in\;grains\;after\;anthesis\left(kg\;ha^{-1}\right)=dry\;weight\;of\;grains\;\\ at\;maturity-assimilates\;transported\;in\;vegetative\;organ\;storage\;before\;anthesis \end{array}$$

#### 2.3.3 Nitrogen accumulation and transport in plants

The N content of dried plant samples was determined using the semi-micro Kjeldahl N determination method after they were crushed with a plant crusher and passed through a 100-mesh sieve. The following were the calculation formula for the related indicators of N absorption, accumulation, distribution, and utilization of winter wheat:


(6) $$N\;accumulation\;\left(kg\;ha^{-1}\right)=N\;content\;\times Dry\;matter\;accumulation$$

(7) $$\begin{array}{c} N\;transport\;before\;anthesis\;\left(kg\;ha^{-1}\right)=N\;accumulation\;in\\ \;vegetative\;organs\;during\;anthesis-N\;accumulation\;in\;vegetative\;organs\;during\;maturity \end{array}$$

(8) $$N\;transport\;efficiency\;before\;anthesis\;\left(\%\right)=\frac{N\;transport\;amount\;before\;anthesis}{N\;accumulation\;during\;anthesis\;period}$$

(9) $$Contribution\;rate\;of\;before\;anthesis\;N\;to\;grain\;N\;\left(\%\right)=\frac{Before\;anthesis\;N\;transport\;amount}{Grain\;N\;accumulation\;amount}$$

(10) $$\begin{array}{c} N\;accumulation\;after\;anthesis\;\left(kg\;ha^{-1}\right)=N\;accumulation\;in\;grains\\ -N\;trasnport\;before\;anthesis \end{array}$$

(11) $$Contribution\;rate\;of\;post\;anthesis\;N\;to\;grain\;N\;\left(\%\right)=\frac{Post\;anthesis\;N\;accumulation}{Grain\;N\;accumulation\;}$$

The calculation formula for NUE related indicators is as follows:


(12) $$N\;absorption\;efficeincy\;\left(NUpE,\;kg\;kg^{-1}\;\right)=\frac{Plant\;N\;accumulation}{N\;application\;rate}$$

(13) $$N\;use\;efficeincy\;\left(NUE,\;kg\;kg^{-1}\;\right)=\frac{Grain\;Yield}{Aboveground\;N\;accumulation\;}$$

(14) $$N\;fertilizer\;partial\;productivity\;\left(PFP,\;kg\;kg^{-1}\;\right)=\frac{Grain\;yield}{N\;application\;rate\;}$$

#### 2.3.4 Yield and its components

Plants were harvested at the maturity stage from a 2.0 m^2^ area from each plot. After threshing, impurities were removed, and the grains were air-dried, and adjusted standard moisture content (12%) to calculate the grain yield per hectare. In each plot, a sample of 1.0 m^2^ area is used for calculating number of spikes per unit area and grains number per spike. The 1000-grain weight was calculated from 1000 grains of the yield measurement samples taken for each plot to measure the yield components.

### 2.4 Statistical analysis

The data were recorded, analyzed and plotted using Microsoft Excel 2016, IBM SPSS 16.0 ((https://www.ibm.com/products/spss-statistics) and Origin 2023b (https://www.originlab.com/index.aspx?go=PRODUCTS/Origin), respectively. Duncan’s test was used for multiple comparisons (P < 0.05). The structural equation (SEM) model is an extension of factor analysis used to test entity theory from empirical data. It is an advanced and robust multivariate statistical method for testing complex path relationship networks. This study assumes that CRNF and ONF application directly or indirectly affect yield components; it was conducted using the ‘plspm’ package of R software, version 3.5.1. model, and report the standardization coefficient of each path in each component model. Significance tests were conducted based on the path coefficients between latent variables.

## 3 Results

### 3.1 Soil nitrogen dynamics

#### 3.1.1 Nitrate nitrogen

**Figure 2:**
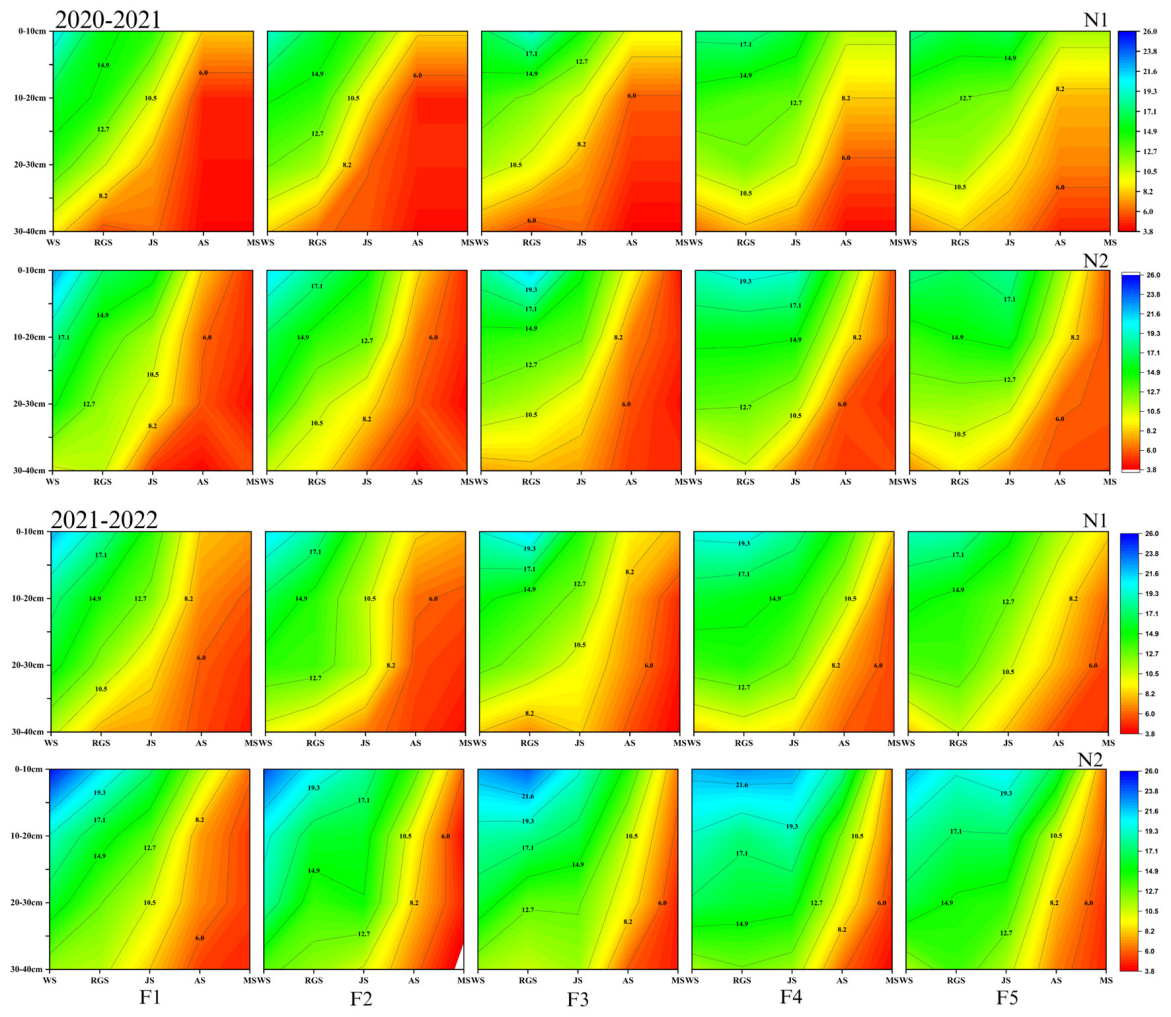
Dynamics of soil nitrate nitrogen (NO_3_^–^N) concentrations in the 0-40 cm soil layer under various combinations of ONF and CRNF treatments at before wintering stage (WS), regreening stage (RGS), jointing stage (JS), anthesis stage (AS) and maturity stage (MS) for the winter wheat planted a) 2020-2021 and b) (2021-2022) growing season. CRNF and ONF are set under two N application rates N_1_ (192 kg ha^-1^) N_2_ (240 kg ha^-1^) of ONF (F_1_) 100% ONF, (F_2_) 70% ONF + 30% CRNF, (F_3_) 50% ONF + 50% CRNF, (F_4_) 30% ONF + 70% CRNF, and (F_5_) 100% CRNF.

Nitrate N is essential for amino acid synthesis, protein production, and promotes tillering in winter wheat. The CRNF fertilizer treatments significantly increased soil N availability. Compared to N_1_, the N_2_ application rate for soil nitrate N was significantly higher at the greening, jointing, and anthesis stages in the 0-20 cm soil layer. During the wintering period, under various CRNF treatments, the F_1_ and F_2_ treatments had significantly higher nitrate N content in the 0-40 cm soil layer (**Figure 2**). At the anthesis and maturity stages, the F_1_, F_2_, and F_3_ treatments were significantly lower than the F_4_ and F_5_ treatments. In 2021-2022, there were no significant differences in the nitrate N content in the 0-40 cm soil layer between treatments (**Figure 2**). Notably, the proportion of CRNF with F_4_ provides a more sustained supply of soil nitrate N from the regreening stage to the anthesis stage of winter wheat compared to the sole application of ONF.

#### 3.1.2 Ammonium nitrogen

**Figure 3:**
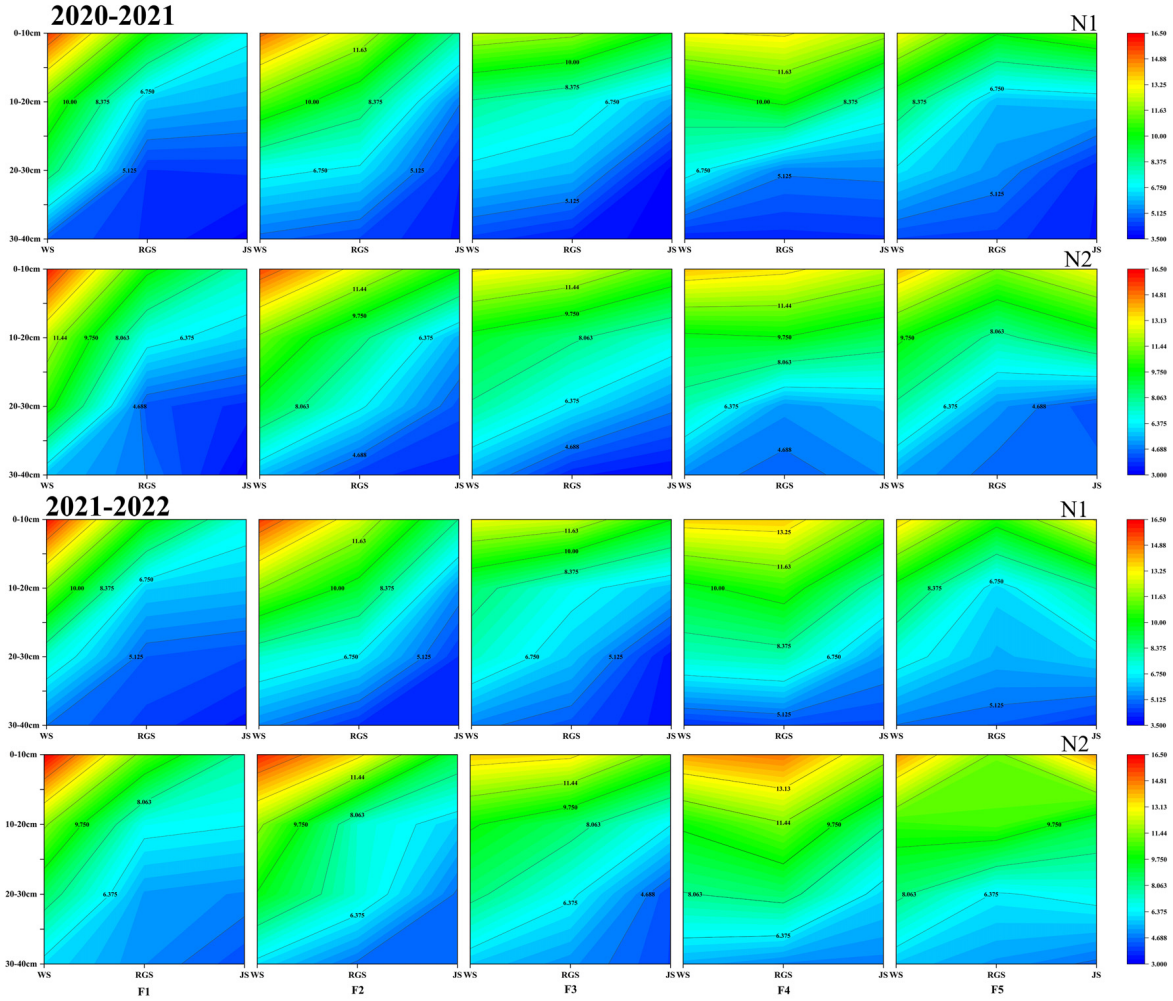
Dynamics of soil ammonium N (NH_4_^+^-N) concentration in 0-40 cm soil layer under various combinations of ONF and CRNF treatments at before wintering stage (WS), regreening stage (RGS) and jointing stage (JS) for the winter wheat planted a) 2020-2021 and b) (2021-2022) growing season. CRNF and ONF are set under two N application rates N_1_ (192 kg ha^-1^) N_2_ (240 kg ha^-1^) of ONF (F_1_) 100% ONF, (F_2_) 70% ONF + 30% CRNF, (F_3_) 50% ONF + 50% CRNF, (F_4_) 30% ONF + 70% CRNF, and (F_5_) 100% CRNF.

Ammonium N promotes and stimulates root growth and development, enhancing water and nutrient uptake. The soil ammonium N content in the 0-40 cm soil layer was lower in 2020-2021 than in 2021-2022 (**Figure 3**). There was no significant difference in ammonium N content between the two N application rates at the wintering, regreening, and jointing stages. When the proportion of CRNF was increased to 70%, higher ammonium N content was observed at the regreening and jointing stages in the 0-20 cm soil layer among different CRNF treatments (**Figure 3**). At jointing stage, the performance of ammonium N in soil was ranked as follow: F_4_> F_3_, F_5_> F_1_, F_2_. The results show that the ammonium N content in 0-40 cm soil is generally lower than the nitrate N content, as the proportion of CRNF increases in the winter wheat regreening and jointing stages.

#### 3.1.3 Soil nitrate nitrogen at maturity stage

**Figure 4:**
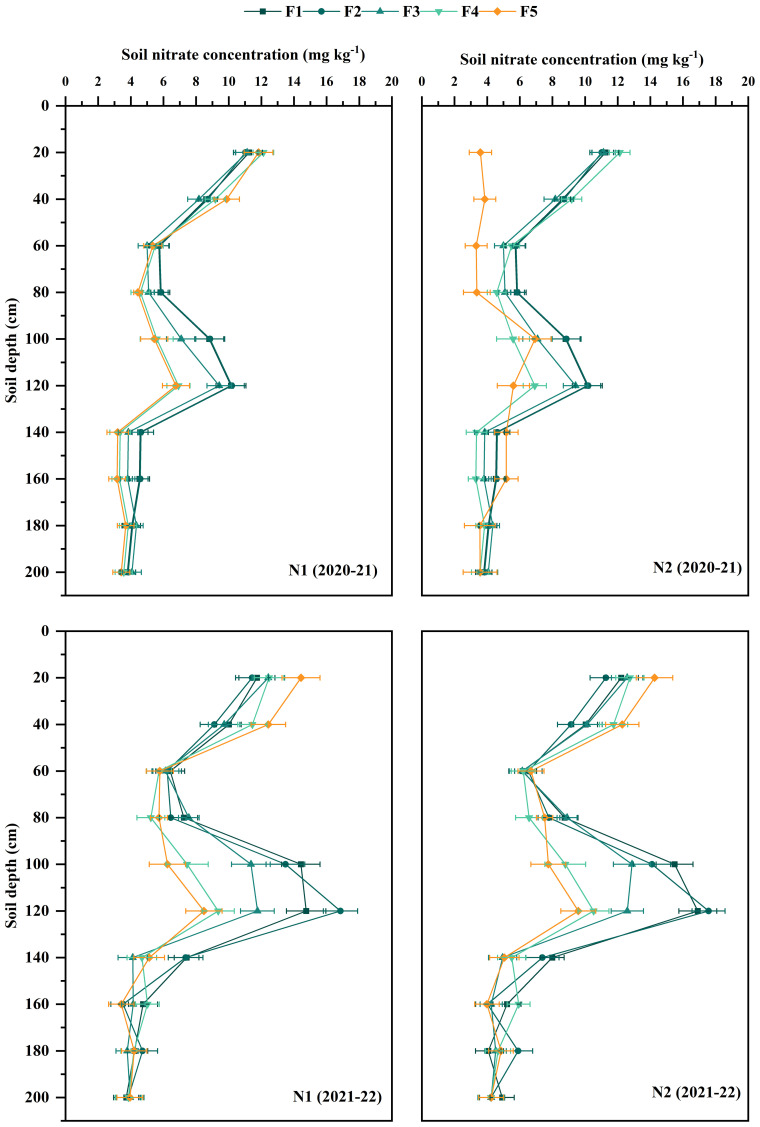
Nitrate N concentration in 0-200 cm soil layer at maturity stages under various combinations of ONF and CRNF treatments for the winter wheat planted (2020-2022) growing season. CRNF and ONF are set under two N application rates N_1_ (192 kg ha^-1^) N_2_ (240 kg ha^-1^) of ONF (F_1_) 100% ONF, (F_2_) 70% ONF + 30% CRNF, (F_3_) 50% ONF + 50% CRNF, (F_4_) 30% ONF + 70% CRNF, and (F_5_) 100% CRNF.

The soil nitrate N content at 0-100 cm during the winter wheat maturity period in 2021-2022 was generally higher than in 2020-2021 (**Figure 4**). The nitrate N accumulation in the 100-200 cm soil layer was significantly higher in the N_2_ application of N rates, when the proportion of CRNF is 0%, 30%, and 50%. The nitrate N accumulation in the 100-200 cm soil layer showed no significant difference between N_1_ and N_2_ application rates when the proportion of CRNF is 70% and 100% (**Figure 4**). Overall, nitrate levels are relatively low, but notable increases are observed between 80-120 cm depth, likely originating from previous seasons’ excessive rainfall and irrigation. The results showed that increasing the amount of ONF applied promotes downward leaching of soil nitrate N, which is detrimental to winter wheat absorption and utilization. CRNF leaches less soil nitrate N and can provide more soil N supply than ONF due to its slower release rate in the early stage. The amount of CRNF applied should be increased appropriately.

### 3.2 Dry matter accumulation and transport

#### 3.2.1 Dry matter accumulation dynamics

**Figure 5:**
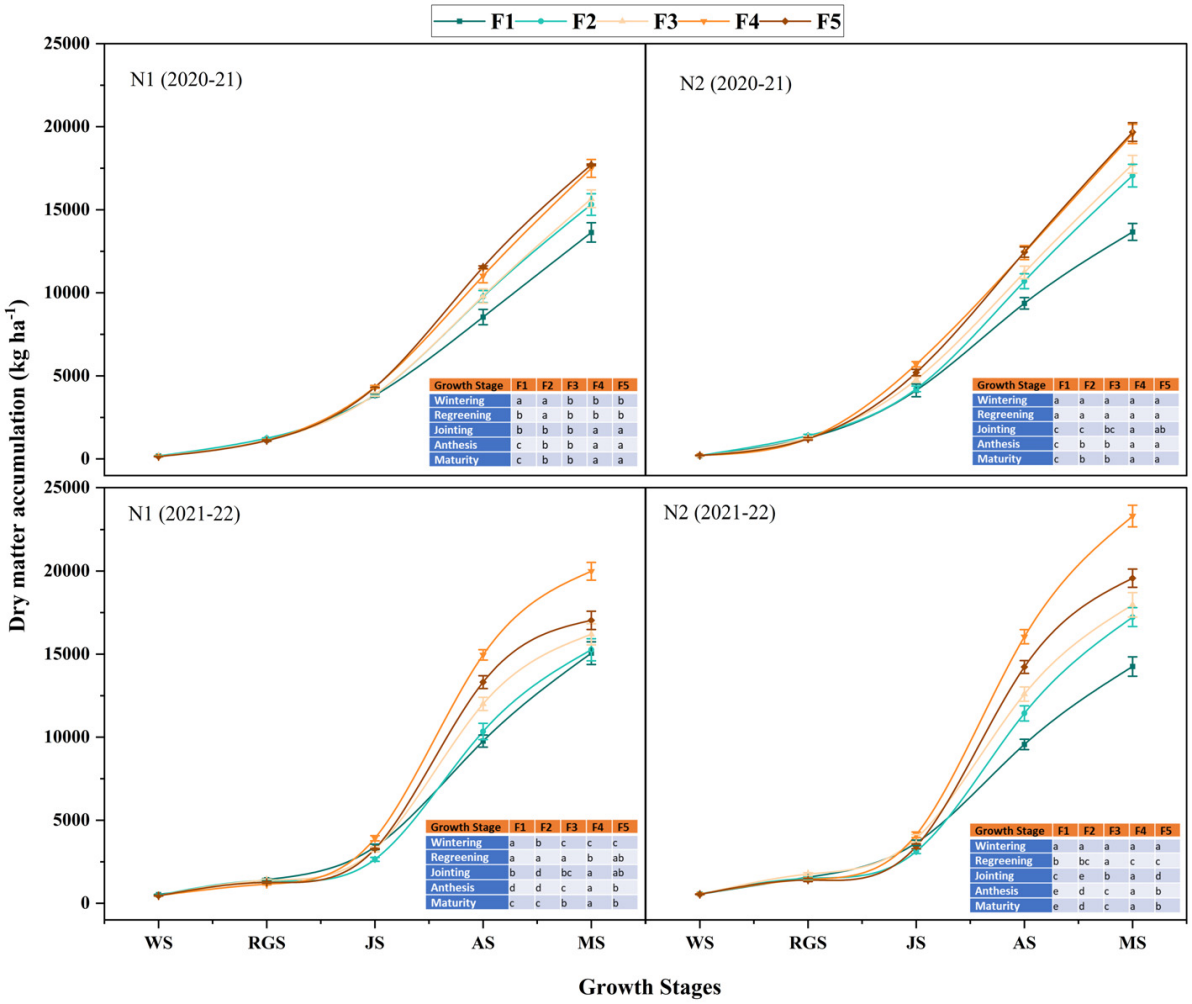
Dynamic changes of dry matter accumulation amounts of winter wheat under various combinations of ONF and CRNF treatments at before wintering stage (WS), regreening stage (RGS) and jointing stage (JS) for the winter wheat planted a) 2020-2021 and b) (2021-2022) growing season. Small letters indicate a significant level of 5% difference. CRNF and ONF are set under two N application rates N_1_ (192 kg ha^-1^) N_2_ (240 kg ha^-1^) of ONF (F_1_) 100% ONF, (F_2_) 70% ONF + 30% CRNF, (F_3_) 50% ONF + 50% CRNF, (F_4_) 30% ONF + 70% CRNF, and (F_5_) 100% CRNF.

Dry matter (DM) accumulation and transport play pivotal roles in winter wheat development and yield, progressively increasing throughout the growth period. The DM accumulation in 2021-2022 was overall higher than that in 2020-2021. Under the two ONF application rates, the N_2_ nitrogen application rate was significantly more significant than the N_1_ nitrogen application rate during the entire growth stages of winter wheat (**Figure 5**). For the proportions of CRNF, there was no significant difference between the blending ratio treatments at the wintering and greening stage; the DM accumulation of winter wheat was higher in the F_2_ and F_3_ treatments than in the other treatments. At the jointing stage, the N_1_ ONF application rate and the F_4_ and F_5_ treatments were significantly higher than others. Under the N_2_ ONF application rate, the F_4_ treatment was significantly higher than the other treatments. At the anthesis and maturity stages, the differences in DM accumulation between different treatments were consistent with the dynamics of the winter wheat population. As the proportion of CRNF increases, the DM accumulation of winter wheat at the anthesis and maturity stages increases. It shows that ONF treatment has obvious advantages before the jointing stage. In contrast, applying CRNF has increased DM accumulation at the anthesis and maturity stages.

#### 3.2.2 Dry matter distribution in various organs

**Figure 6:**
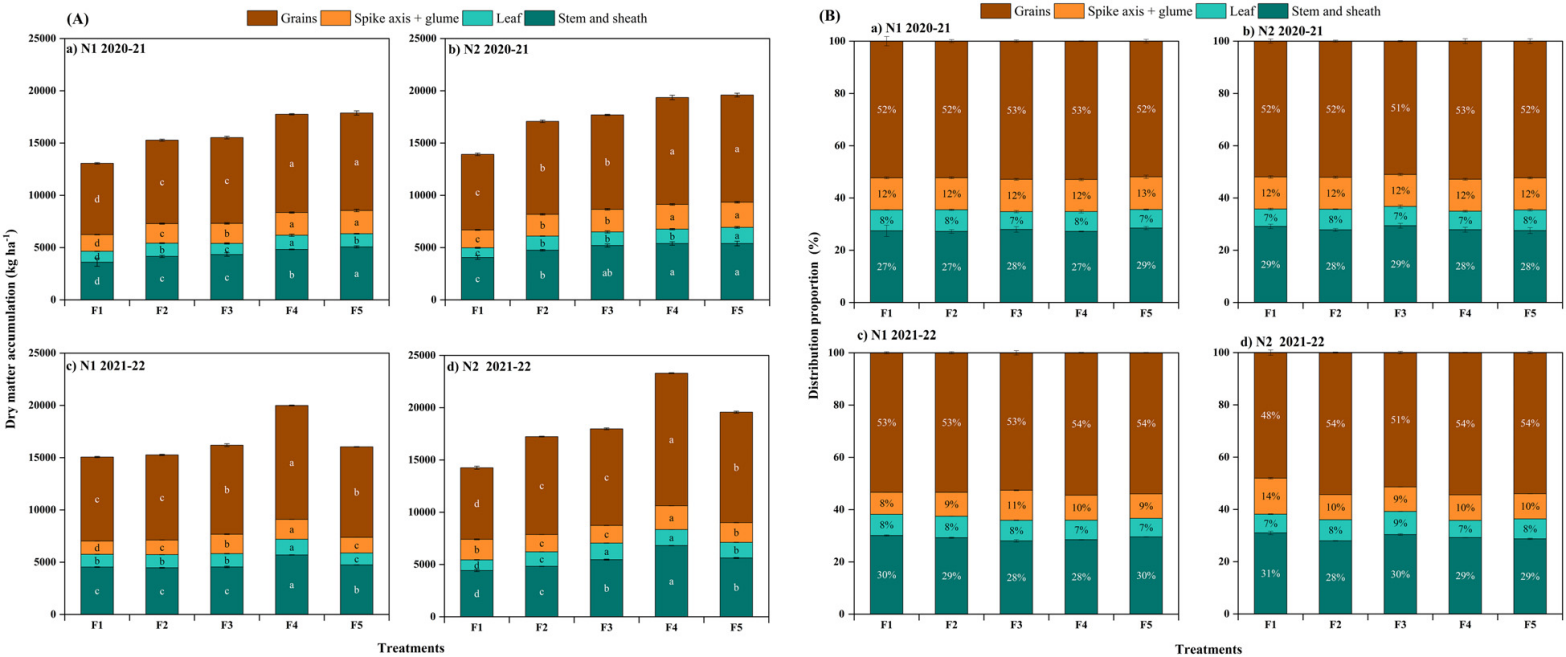
Plant dry matter accumulation **(A)** and distribution proportion **(B)** in different organs of winter wheat under various combinations of ONF and CRNF treatments at anthesis stage for the winter wheat planted in 2020-2021 and 2021-2022 growing seasons. Small letters indicate a significant level of 5% difference; CRNF and ONF are set under two N application rates N_1_ (192 kg ha^-1^) N_2_ (240 kg ha^-1^) of ONF (F_1_) 100% ONF, (F_2_) 70% ONF + 30% CRNF, (F_3_) 50% ONF + 50% CRNF, (F_4_) 30% ONF + 70% CRNF, and (F_5_) 100% CRNF.

The ranking of DM accumulation and distribution ratio among different organs at maturity for both years was as follows: grain > stem and sheath > spike axis and glume > leaf (**Figure 6A**). When comparing the F_4_ treatment of CRNF to the other treatments, the DM accumulation of grains at the maturity stage was significantly higher in the F_4_ treatment. The DM content in the stem + leaf sheath, spike axis + glume, and leaves was significantly higher in the F_4_ treatment (**Figure 6**). It suggests that increasing the amount of N application can significantly increase the DM distribution of each organ during the maturity stage without significant changes in the distribution ratio (**Figure 6B**). A high proportion of CRNF is beneficial for increasing DM accumulation in each organ during the maturity stage, which is helpful for winter wheat.

The translocation amount, transport efficiency, and contribution rate of assimilates stored in vegetative organs before anthesis of winter wheat in 2021-2022 are significantly higher than in 2020-2021, while post-anthesis assimilates in the grains and their contribution rate to the grains was significantly lower in 2020-2021 (**Table 2**). The results show that increasing the amount of N can increase the distribution of assimilates in the grains after anthesis; appropriately increasing the proportion of CRNF can increase the assimilates amount stored in the vegetative organs before anthesis and the distribution of assimilates in the grains after anthesis. There is no significant difference in the contribution rate of DM to grains before and after anthesis. Among CRNF treatments, the transfer amount of assimilates stored in vegetative organs before anthesis was significantly higher in the F_4_ and F_5_ treatments than in other treatments; the distribution amount of assimilates in the grains after anthesis was significantly higher in the F4 treatment.

**Table 2:** Dry matter assimilation and its contribution to grain of winter wheat after anthesis under different treatments.

Treatment	Pre-anthesis			Post anthesis	
	Translocation	Translocation	Contribution	Translocation	Contribution
	Amount (kg ha^-1^)	(%)	(%)	Amount (kg ha^-1^)	(%)
**2020-2021**	2710.8b	25.38b	31.23b	6024.4a	68.77a
**2021-2022**	4234.0a	33.37a	45.10a	5060.3b	54.90b
**N_1_**	3502.7a	30.61a	40.28a	5094.4b	59.72a
**N_2_**	3442.1a	28.13a	36.05a	5990.4a	63.95a
**F_1_**	2470.2b	26.43b	34.18a	4761.9c	65.82a
**F_2_**	2947.5b	27.71ab	34.28a	5648.3b	65.72a
**F_3_**	3313.2b	28.72ab	37.91a	5434.4bc	62.09a
**F_4_**	4313.3a	30.93ab	39.30a	6486.9a	60.70a
**F_5_**	4317.9a	33.07a	45.15a	5380.5bc	54.85a
N	ns	ns	ns	**	ns
F	**	ns	ns	**	ns
N×F	ns	ns	ns	ns	ns

CRNF and ONF are set under two N application rates N_1_ (192 kg ha^-1^) N_2_ (240 kg ha^-1^) of ONF (F_1_) 100% ONF, (F_2_) 70% ONF + 30% CRNF, (F_3_) 50% ONF + 50% CRNF, (F_4_) 30% ONF + 70% CRNF, and (F_5_) 100% CRNF. Different letters indicate the significance within the same year at 5% level by LSD test. ns: not significant, (p > 0.05); **: Significant at p < 0.01.

### 3.3 Nitrogen accumulation and transport

#### 3.3.1 Nitrogen accumulation dynamics

**Figure 7:**
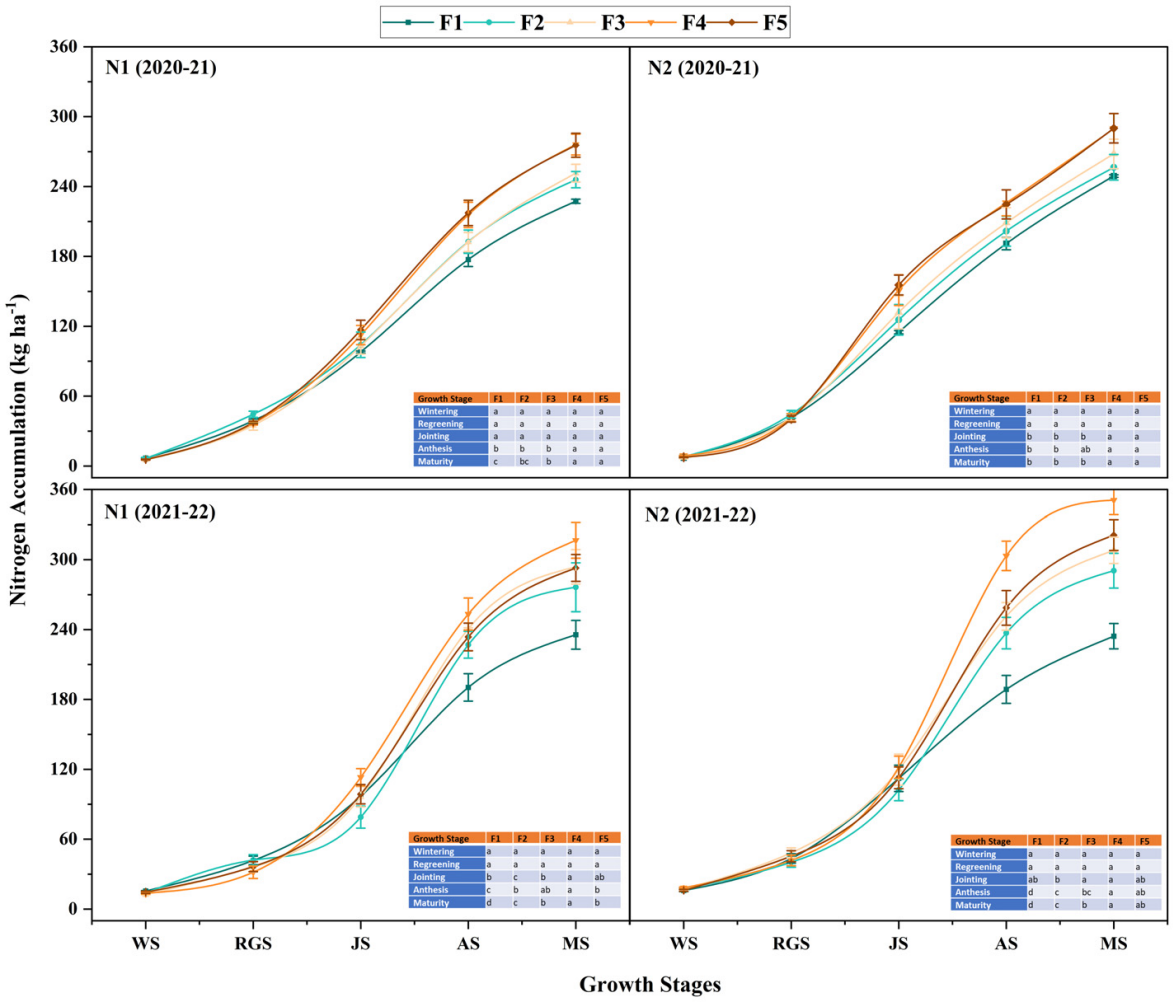
Dynamic changes of nitrogen accumulation amount of winter wheat under various combinations of ONF and CRNF treatments at before wintering stage (WS), regreening stage (RGS) and jointing stage (JS) for the winter wheat planted a) 2020-2021 and b) (2021-2022) growing season. Small letters indicate a significant level of 5% difference; CRNF and ONF are set under two N application rates N_1_ (192 kg ha^-1^) N_2_ (240 kg ha^-1^) of ONF (F_1_) 100% ONF, (F_2_) 70% ONF + 30% CRNF, (F_3_) 50% ONF + 50% CRNF, (F_4_) 30% ONF + 70% CRNF, and (F_5_) 100% CRNF.

Under different N application rates, N accumulation at different growth stages in 2021-2022 was significantly higher than in 2020-2021. During winter wheat’s anthesis and maturity stages, the treatment N_2_ accumulated more N than N_1_ (**Figure 7**). There was no significant difference between treatments with different proportions of CRNF at the wintering, regreening, and jointing stages (**Figure 7**). At the anthesis and maturity stages, the F_4_ treatment was significantly higher than other treatments, and the overall performance was F_4_>F_5_, F_3_>F_2_, and F_1_, with significant differences between treatments. The rules for each treatment’s blending ratio were consistent with the anthesis stage. The results showed that increasing the amount of N applied can increase the N accumulation in winter wheat at the anthesis and maturity stages.

#### 3.3.2 Nitrogen distribution in various organs

**Figure 8:**
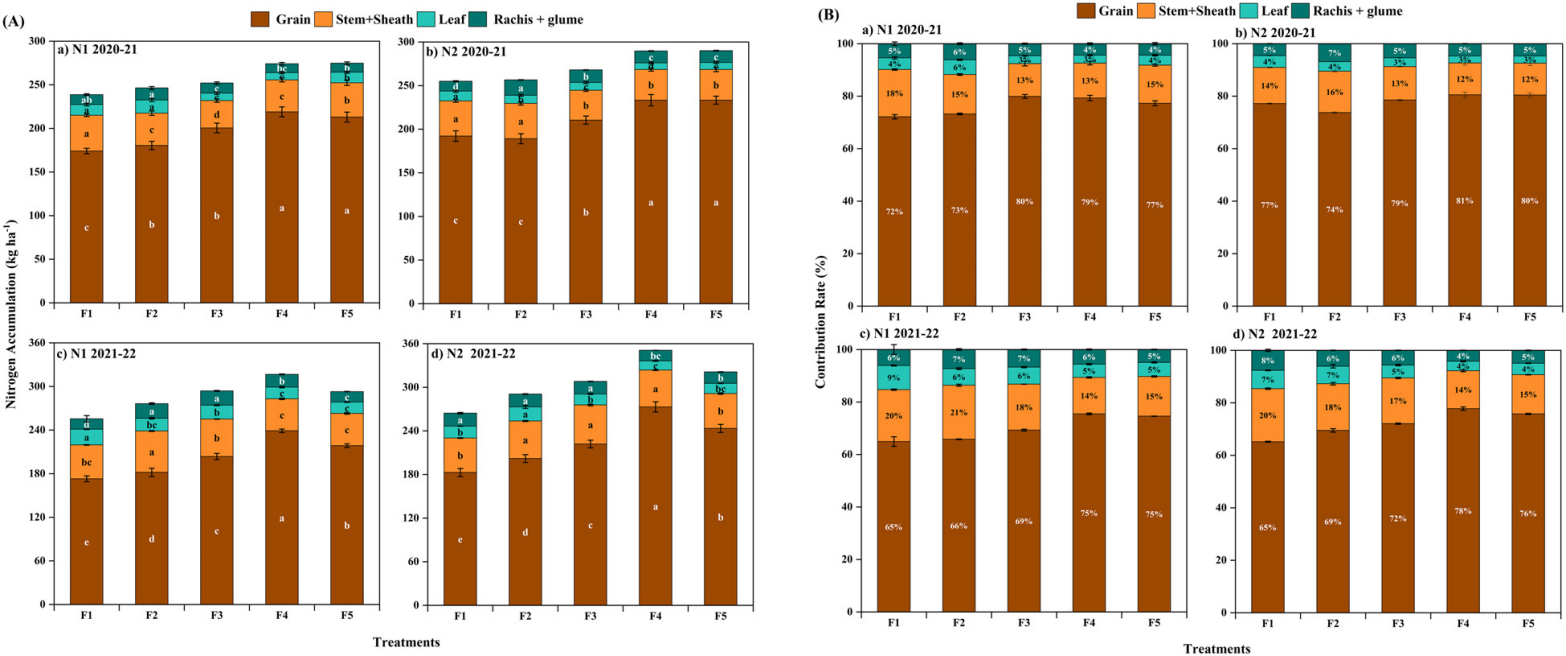
Nitrogen accumulation **(A)** and distribution proportion **(B)** in different organs of winter wheat under various combinations of ONF and CRNF treatments at anthesis stage for the winter wheat planted in 2020-2021 and 2021-2022 growing season. Note: Small letters indicate a significant level of 5% difference; CRNF and ONF are set under two N application rates N_1_ (192 kg ha^-1^) N_2_ (240 kg ha^-1^) of ONF (F_1_) 100% ONF, (F_2_) 70% ONF + 30% CRNF, (F_3_) 50% ONF + 50% CRNF, (F_4_) 30% ONF + 70% CRNF, and (F_5_) 100% CRNF.

The N accumulation in mature winter wheat grains did not differ significantly between the two years; however, the N distribution amount and distribution ratio of the stem + sheath, rachis + glume, and leaves were significantly higher in 2021-2022 (**Figure 8A**). The amount of N distributed in the grains at maturity was significantly higher in the F4 treatment of CRNF. The overall performance was F_4_>F_5_>F_3_>F_2_>F_1_, with significant differences between treatments (**Figure 8**). The results showed that appropriately increasing the amount of N can increase the amount of N distributed and the distribution ratio in winter wheat grains during the maturity stage while decreasing the amount of N distributed and the distribution ratio in leaves (**Figure 8B**). Increasing the proportion of CRNF improves the N distribution of vegetative organs. The grain N accumulation and distribution ratio reaches its maximum when the proportion of CRNF is at 70%.

#### 3.3.3 Nitrogen redistribution

The contribution rate of post-anthesis N to grain N, the transport efficiency of pre-anthesis N in winter wheat, and the contribution of pre-anthesis N to grain N were given in **Table 3**. The pre-anthesis N transfer amount and post-anthesis N accumulation amount of N application N_2_ were significantly higher than those of N application N_1_. The amount of pre-anthesis N transport in the F_4_ treatment of CRNF was significantly higher than others. The overall performance was F_4_>F_5_>F_3_>F_2_>F_1_, and have significant differences between treatments (**Table 3**). The contribution rate of pre-anthesis N to grain N was significantly lower in the F_1_ treatment. The F_1_ treatment has the lowest post-anthesis N accumulation, and the F_1_ treatment has a significantly higher post-anthesis N contribution rate to the grains. The results show that increasing the N application rate can increase the N accumulation in winter wheat grains during maturity by increasing pre-anthesis N transport and post-anthesis N accumulation. Increasing the grain accumulation of winter wheat at maturity relied on enhancing both the amount and efficiency of N transport before anthesis.

**Table 3:** Effects of different ONF and CRNF treatments on nitrogen redistribution in winter wheat plants.

	Pre-anthesis			Post anthesis	
	NRA (kg ha^-1^)	NRR (%)	NRCT (%)	NAG (kg ha^-1^)	NACT (%)
**2020-2021**	145.34a	70.76a	71.32b	58.16a	28.68a
**2021-2022**	152.89a	64.42b	72.99a	56.01a	27.01b
**N_1_**	145.34b	66.35a	71.93a	55.12b	28.07a
**N_2_**	152.89a	68.83a	72.38a	59.05a	27.62a
**F_1_**	115.74e	61.94c	69.97b	49.73c	30.03a
**F_2_**	135.45d	63.32c	71.89a	52.88b	28.11b
**F_3_**	152.22c	68.61b	72.75a	56.92ab	27.25b
**F_4_**	176.42a	73.06a	73.15a	64.63a	26.85b
**F_5_**	165.75b	71.03ab	73.00a	61.27ab	27.00b
N	**	**	ns	ns	ns
F	**	**	ns	ns	ns
N×F	ns	**	ns	ns	ns

NRA, N remobilization amount in vegetative organs; NRR, N remobilization ratio in vegetative organs; NRCT, total contribution rate of N remobilization pre-anthesis to grain N; NAG, N uptake amount after anthesis; NACT, total contribution rate of N accumulated post-anthesis to the grain N. CRNF and ONF are set under two N application rates N_1_ (192 kg ha^-1^) N2 (240 kg ha^-1^) of ONF (F_1_) 100% ONF, (F_2_) 70% ONF + 30% CRNF, (F_3_) 50% ONF + 50% CRNF, (F_4_) 30% ONF + 70% CRNF, and (F_5_) 100% CRNF. Different alphabets indicate the significance within the same year at 5% level by LSD test. ns: not significant, (p > 0.05); **: Significant at p < 0.01.

### 3.4 Nitrogen utilization efficiency

**Figure 9:**
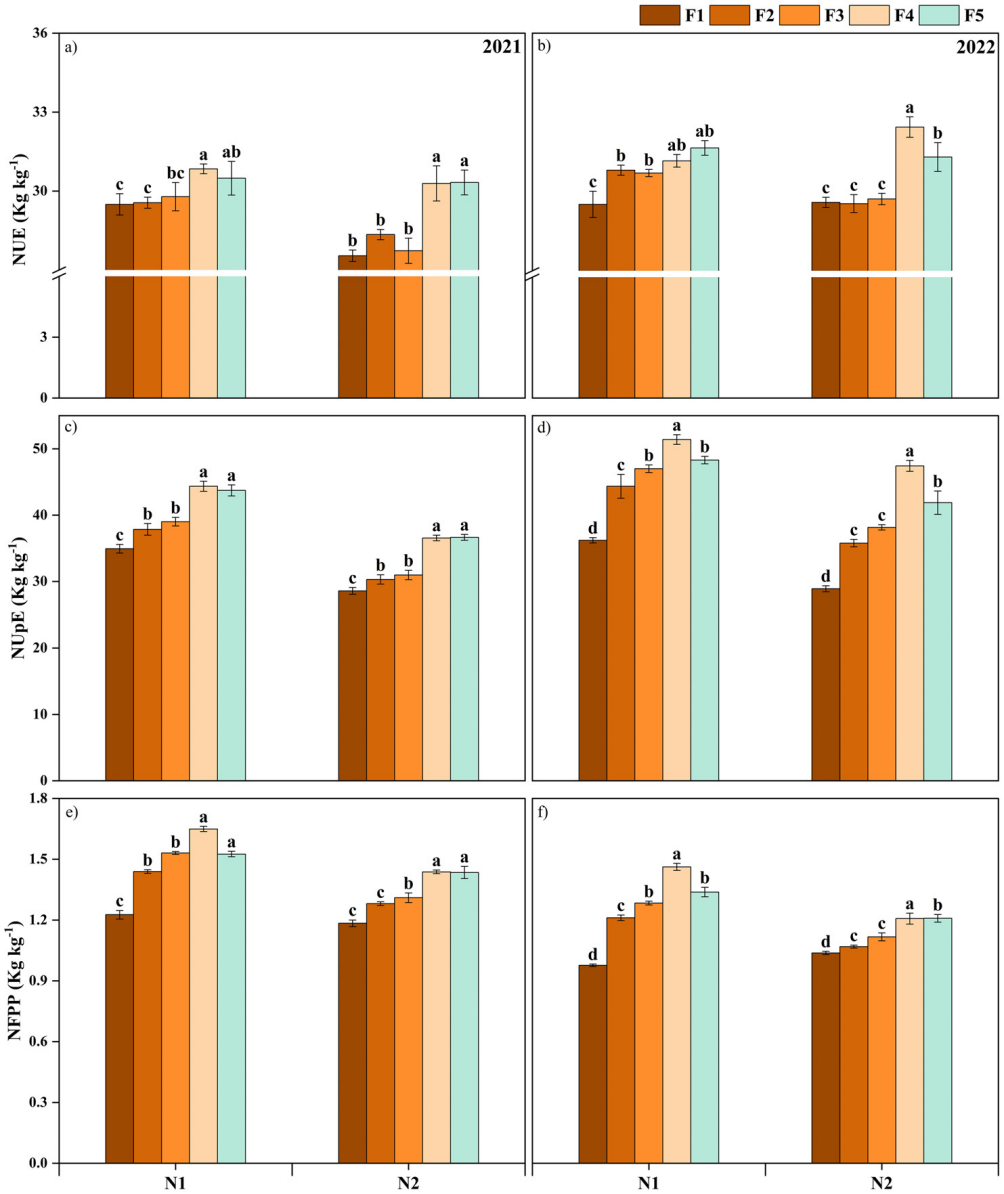
Nitrogen use efficiency (a, b) nitrogen uptake efficiency (c, d) nitrogen fertilizer partial productivity (e, f); winter wheat under various combinations of ordinary nitrogen fertilizer and controlled released nitrogen fertilizer treatments for the winter wheat planted 2020-2022 growing season. CRNF and ONF are set under two N application rates N1 (192 kg ha-1) N2 (240 kg ha-1) of ONF (F1) 100% ONF, (F2) 70% ONF + 30% CRNF, (F3) 50% ONF + 50% CRNF, (F4) 30% ONF + 70% CRNF, and (F5) 100% CRNF. Vertical bars are means ± standard deviation. Different letters mean that the difference of the means between treatments was significant (LSD test, P < 0.05).

The N use efficiency (NUE), N uptake efficiency (NUpE), and N fertilizer partial productivity (NFPP) in 2021-2022 are significantly higher than that in 2020-2021. The NUE revealed no significant difference in N_1_ and N_2_ application amounts of ONF when the proportion of CRNF was F_4_ (70%) and F_5_ (100%) (**Figure 9**). For both years, the CRNF treatment F_4_ combined with ONF at N_2_ had the highest NUpE and NFPP, significantly higher than the other treatments. There was no significant difference in NUE between N_1_ and N_2_ of ONF for the CRNF for F_4_ treatment, although when the N application amount increased, the NUE did not change significantly. Appropriately increasing the proportion of CRNF can improve winter wheat’s NUE, NUpE, and NFPP in comparison to the F_1_ of CRNF; the NUE of the F_4_ treatment increased by an average of 2.14% under the N_2_ application of ONF in both years.

### 3.5 Yield and its components

Winter wheat yield and its components were significantly enhanced by the combination of CRNF with ONF for both years (p < 0.05). Compared to 2020-2021, the number of spikes, grains per spike, and grain yield in 2021-2022 were significantly higher, while the 1000-grain weight was higher in 2020-2021 than in 2021-2022 (**Table 4**). Winter wheat yield gradually increased in all blended treatments of CRNF. There was no significant difference in the number of grains per spike or 1000-grain weight between the two N application rates when the proportion of CRNF was 0%, 30%, or 50%. The results showed that appropriately increasing the amount of applied N can increase grain yield by increasing the number of spikes. Compared to other fertilizer applications, the maximum number of spikes, grains per spike, and grain yield were observed with treatment of CRNF 70% combined with N_2_ of ONF.

**Table 4:** Winter wheat grain yield and its components under various treatments of ONF combined with CRNF.

Treatment		Number of spikes (× 10^4^ ha^-1^)	2020-2021		Grain yield (kg ha^-1^)	Number of spikes (× 10^4^ ha^-1^)	2021-2022		Grain yield (kg ha^-1^)
			Grains per spike	1000-Grain weight (g)			Grains per spike	1000-Grain weight (g)	
**N_1_**	**F_1_**	431.33 ± 21.36c	34.60 ± 0.58a	49.7 ± 0.30ab	6705.97 ± 71.77c	473.98 ± 3.54d	35.93 ± 2.35b	48.02 ± 0.45a	6950.50 ± 44.49d
	**F_2_**	469.33 ± 4.25b	36.90 ± 1.02a	49.1 ± 0.30b	7268.17 ± 97.45b	543.32 ± 4.22c	37.13 ± 0.67ab	46.64 ± 0.76a	8547.33 ± 198.76c
	**F_3_**	475.33 ± 4.37b	36.56 ± 1.82a	50.1 ± 0.48ab	7489.17 ± 72.19b	553.74 ± 9.03bc	39.06 ± 1.14ab	46.44 ± 0.13a	8989.77 ± 62.85b
	**F_4_**	506.00 ± 7.54ab	35.70 ± 1.12a	50.6 ± 0.60a	8513.90 ± 84.32a	599.60 ± 2.07a	39.76 ± 0.90a	47.08 ± 1.52a	9867.83 ± 80.69a
	**F_5_**	515.66 ± 2.33a	36.40 ± 0.75a	49.7 ± 0.55ab	8395.03 ± 92.20a	566.95 ± 1.67b	39.73 ± 0.40a	47.83 ± 0.76a	9271.81 ± 62.58b
**N_2_**	**F_1_**	446.33 ± 8.29c	36.36 ± 1.47a	48.5 ± 0.29a	6857.30 ± 69.49c	463.69 ± 9.95e	37.83 ± 1.38a	46.17 ± 0.20a	6934.97 ± 62.48d
	**F_2_**	500.00 ± 1.00b	34.13 ± 0.73a	48.8 ± 0.08a	7271.83 ± 97.65b	546.52 ± 2.32d	37.56 ± 0.61a	47.72 ± 0.20a	8800.20 ± 77.12c
	**F_3_**	504.00 ± 1.73b	34.50 ± 0.43a	49.4 ± 0.05a	7432.00 ± 97.04b	583.20 ± 5.55c	39.43 ± 0.68a	46.46 ± 0.94a	8940.87 ± 55.17c
	**F_4_**	536.66 ± 5.69a	34.83 ± 0.98a	49.7 ± 0.41a	8770.87 ± 58.81a	639.20 ± 1.83a	39.86 ± 0.41a	45.98 ± 0.07a	11381.80 ± 114.38a
	**F_5_**	541.00 ± 2.88a	35.63 ± 0.49a	49.6 ± 0.46a	8793.57 ± 61.41a	605.70 ± 5.27b	40.00 ± 1.56a	46.65 ± 0.27a	10051.63 ± 243.67b
N		*	ns	ns	ns	*	ns	ns	ns
F		**	ns	ns	**	**	ns	ns	**
N×F		ns	ns	ns	ns	ns	ns	ns	ns

CRNF and ONF are set under two N application rates N_1_ (192 kg ha^-1^) N_2_ (240 kg ha^-1^) of ONF (F_1_) 100% ONF, (F_2_) 70% ONF + 30% CRNF, (F_3_) 50% ONF + 50% CRNF, (F_4_) 30% ONF + 70% CRNF, and (F_5_) 100% CRNF. Different alphabets indicate the significance within the same year at 5% level by LSD test. ns: not significant, (p > 0.05); *: Significant at p < 0.05; **: Significant at p < 0.01.

### 3.6 Relationship between nitrogen fertilizer dynamics to yield and its components

**Figure 10:**
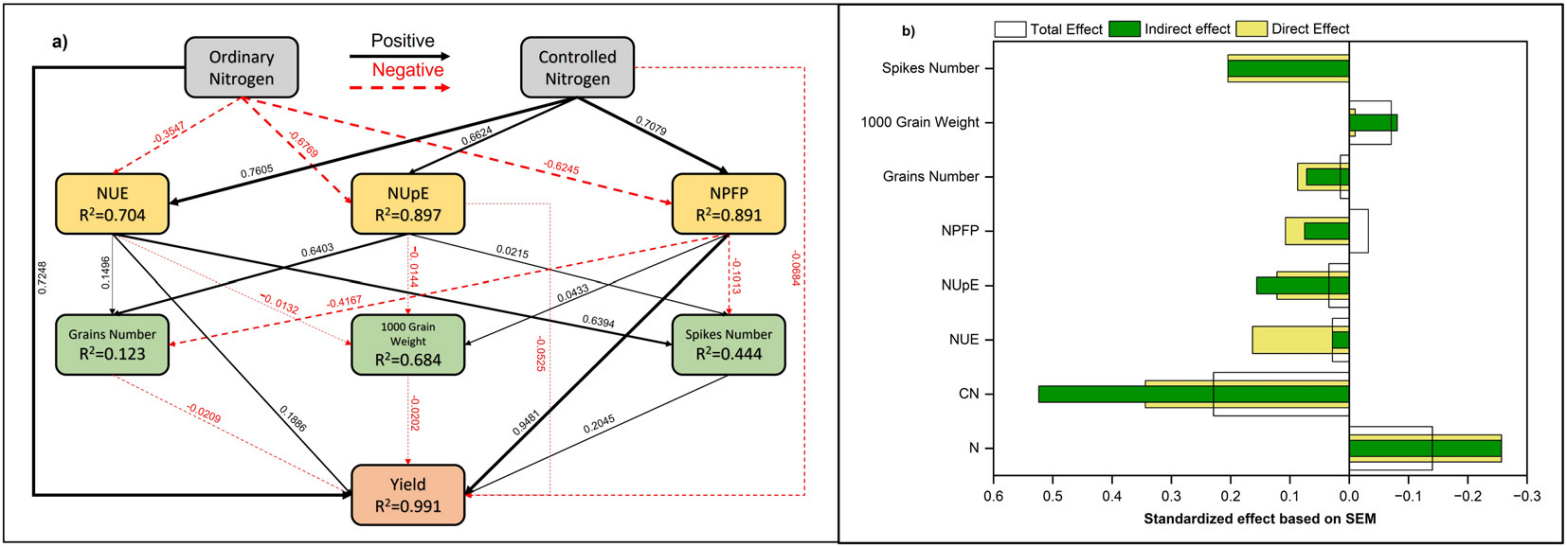
Relationship between ONF and CRNG and yield components of winter wheat. **(A)** The structural equational model (SEM) framework describing ONF and CRNF, NUE, NUpE, NPFP, grain number, 1000 grain weight, spike number, and grain yield. The red dotted lines and black solid lines are indirect and direct effects. Numbers above/below the arrow line indicate correlation. The interpretation variance (R2) ratio appears next to each indicator in the model. **(B)** The direct, indirect, and total effects of different potential variables on winter wheat yield are based on SEM data. N, Ordinary nitrogen; CN, Controlled nitrogen; NUE, Nitrogen use efficiency; NUpE, Nitrogen uptake efficiency; and NPFP, Nitrogen fertilizer partial productivity.

The linear relationship between ordinary N fertilizer (ONF), controlled released N fertilizers (CRNF), N use efficiency (NUE), uptake efficiency (NUpE), partial fertilizer productivity (NPFP) with yield and its components (Grain Number, 1000 Grain Weight, Spikes Number) combined for both the 2020–2022 growing seasons was fitted using structural equation modelling as illustrated in **Figures 10A,B**. The results showed that CRNF has a positive impact on NUE, NUpE, and NPFP with path coefficients (λ) of 0.704, 0.897, 0.891 respectively and indirectly increased winter wheat yield by increasing the number of grains in spikes (**Figure 10**). While, ONF directly affected winter wheat and increased yield (λ: 0.774), while CRNF positively impacted yield (λ: -0.064). The N application also indirectly increased yield by increasing spike number (λ: 0.686). The three N-related indexes have a direct positive effect on winter wheat yield was mainly due to the significant increase in spikes number.

## 4 Discussion

### 4.1 Impact of CRNF on soil nitrogen supply and dry matter accumulation

Nitrogen is essential for plant growth and development, so its availability is critical for crop productivity and applying ONF combined with CRNF offers a simple and efficient framework to improve fertilizer utilization efficiency. Slowly releasing nutrients from the CRNF over time can effectively prevent N accumulation in the soil quickly, reduce nutrient loss to the environment, and thus increase nutrient availability for crops (Vejan et al., 2021; Ma et al., 2021). The results showed that the soil nitrate N and ammonium N content in the jointing and anthesis stages were higher in the CRNF treatments by 70% when combined with the N2 application of ONF. A high amount of CRNF can provide a sufficient supply of soil N in the late growth period of winter wheat. Because the N-release characteristics of CRNF are greatly affected by soil moisture content and temperature, the early nutrient release of CRNF will be too slow, and a one-time basal application will result in insufficient soil nutrient supply in the early stages of winter wheat growth (Geng et al., 2015; Ma et al., 2021). When the proportion of CRNF is 70% and ONF at N2, it can not only ensure the soil nitrate N and ammonium N content in the early growth period of winter wheat but also significantly increase the soil nitrate N and ammonium N content in the jointing stage. In this way, one-time fertilization of CRNF can meet the N demand of crops throughout the growth stages, thereby increasing grain yield and NUE, minimizing N leaching and volatilization losses, and reducing the impact on the environment (Azeem et al., 2014).

In the current study, compared to the application of ONF, the application of CRNF increases the nitrate N accumulation in the 0-40 cm soil layer of winter wheat during the maturity period, and when the proportion of CRNF is 70%, it can reduce nitrate N content in the 60-100 cm soil layer and reducing N leaching. Our study shows no significant difference in the nitrate N content in the 0-40 cm soil layer during the winter wheat maturity stage, while the nitrate N content in the 1-2 m soil layer is significantly higher than that of other treatments (**Figure 4**). The lowest N-releasing proportions were CRNF at 70% and 100%, and there was no significant difference in nitrate N accumulation in the 1-2 m soil layer with ONF N2 application, indicating that winter wheat requires more N in the late growth period. The soil N release in the later growth period of winter wheat is more consistent with the N uptake of winter wheat when the proportion of CRNF is 70%, thereby increasing the N accumulation during the maturity period of winter wheat while reducing N leaching risk.

Nitrogen fertilizer significantly impacts the number of tillers in the early growth stage of winter wheat. Stem and tiller dynamics are essential for regulating winter wheat population dynamics and achieving the maximum yield. Nutrient release is too slow in the early growth stage, but increasing the dosage of CRNF can increase soil nutrients before and after the jointing stage of winter wheat, significantly increasing the number of spikes at maturity (Ma et al., 2021, Ma et al., 2022a, Ma et al., 2022b). The application of CRNF at 70% combined with ONF can significantly increase the DM accumulation of winter wheat from regreening to anthesis stages compared with the other proportions of CRNF. Nishimura et al. (2022) reported a similar finding that CRNF can regulate the population size of winter wheat and increase the number of spikes per plant to varying degrees, thus increasing the number of spikes at maturity. The application of CRNF maintains a higher tiller number in the late growth stage, laying the foundation for increased yield.

Winter wheat yield formation depends on DM accumulation, particularly post-anthesis DM accumulation, which can significantly improve harvest index and grain yield (Ercoli et al., 2008; Lijuan et al., 2023). In this study, CRNF combined with ONF has increased the DM accumulation from the greening to the maturity stages by increasing the population number. The amount of stored assimilates transported by vegetative organs before anthesis increased when the CRNF (70%) was combined with ONF (N2). However, the distribution of DM in the grains following anthesis was significantly reduced (**Table 2**). The distribution amount was significantly higher than that of other treatments. However, there was no significant difference among the blending ratio treatments in the contribution rate of assimilates stored in vegetative organs before anthesis and assimilates after anthesis to grains. It shows that the soil nutrient supply in the treatment with CRNF (70%) is more consistent with the nutrient absorption of winter wheat. The DM accumulation increased to varying degrees during the vegetative and reproductive growth stages, thereby increasing the grain yield of winter wheat. The results are consistent with the findings of Cui et al. (2022).

### 4.2 Impact of CRNF on nitrogen accumulation and transport

Understanding the dynamics of nutrient accumulation in crops is essential to obtain optimum yield. The ability of a crop to accumulate nutrients varies according to the nutrients used. The translocation of pre-anthesis N to the grain is essential for winter wheat to get maximum yield (Xu et al., 2005). Our results showed that the N accumulation of CRNF (100%) combined with ONF (N2) at the greening stage of winter wheat is low. In contrast, the N accumulation increases rapidly after the jointing stage. The N accumulation of winter wheat has been significantly increased at winter wheat’s anthesis and maturity stages by improving the soil N supply at later growth stages. The N accumulation in grains includes the transfer of N from vegetative organs to the grain and N accumulation in grains after anthesis (Kong et al., 2016). Our results align with the previous findings of Abid et al. (2016), which show that increasing soil N during the early growth period increases the N content of vegetative organs. Increasing soil N in the late growth period can improve the distribution of N in grains after anthesis, resulting in higher grain N content (Chen et al., 2015). The N uptake during the maturity stage was significantly lower in combination with CRNF and ONF. As ONF releases fertilizer efficiency quickly, one-time basal fertilization of ONF cannot meet the N requirement in the later crop growth stages (Zhang et al., 2022a). The amount of N released in the early stage of CRNF is small, making N absorption by vegetative organs difficult, which is not conducive to the transport of N to the grain (Ma et al., 2023). Our findings showed that CRNF (70%) combined with ONF (N2) not only ensured the soil N supply during the greening and jointing stages of winter wheat, but also significantly increased the soil N content at the anthesis stage.

In winter wheat, the N uptake by grains has been affected by N accumulation during anthesis. When N accumulation is increased during anthesis, it can promote the transfer of N from pre-anthesis vegetative organs to the grain and inhibit post-anthesis N absorption (Wu et al., 2021). Our results showed that when the N accumulation during the anthesis period is 272.8 kg ha-1, the contribution rate of pre-anthesis N to grain N reaches the maximum. When it is lower than 272.8 kg ha-1, increasing N accumulation during the anthesis period increased the contribution rate of pre-anthesis N to grain N. The treatment with CRNF (70%) combined with ONF (N2) has increased N accumulation during the anthesis stage of winter wheat and significantly increased the transfer of N from vegetative organs to the grain, resulting in higher grain N content during the maturity stage. Although the input of assimilated N into grains after anthesis showed an increasing trend, the contribution rate of post-anthesis N to grain N showed a decreasing trend. It showed that CRNF at 70% combined with ONF (N2) can provide a good supply of soil nutrients during the reproductive growth stage of winter wheat; because winter wheat accumulates more N during the vegetative growth stage, the N in the grain is transported from the vegetative organs.

### 4.3 Impact of CRNF on yield and nitrogen use efficiency

Nitrogen fertilizer application is critical to the growth of winter wheat, and its management is the key to improving crop yields (Duan et al., 2019; Hussain et al., 2023a). Our results showed that increasing soil N supply after the greening stage with CRNF (70%) increased plant populations at the jointing and anthesis stages, significantly increasing wheat spikes at maturity and hence increasing the yield. Our experiment also found that when the proportion of CRNF was 0%, 30%, and 50%, there was no significant difference in yield between ONF 192 kg ha^-1^ and 240 kg ha^-1^. However, when the proportion of CRNF (70%) was combined with ONF (N2), the yield was significantly improved. Our results are in line with Yang et al. (2011) for the winter wheat yield. Compared to the sole ONF, the CRNF treatment has increased the grain yield with an average of 6.64% for both years. CRNF was applied twice (at the sowing and re-greening stage) and synchronized with the N demand of winter wheat, which has several advantages, such as significantly improved grain yield and NUE. It shows that appropriately increasing the proportion of CRNF can further unleash the potential of N fertilizer input to increase production and efficiency and break the bottleneck of increasing N without increasing production to a certain extent.

The judicious application of fertilizers ensures the supply of nutrients required for crop growth and development, reduces fertilizer losses and improves fertilizer use efficiencies (Dimkpa et al., 2020). Because the application cost of CRNF is higher than ONF’s, the cost can be reduced by combining CRNF with ONF, and the benefits of CRNF can be enhanced. In our experiment, CRNF at 70% combined with ONF (N2) application rates significantly improved N accumulation in the aboveground portion, thus improving grain yield and NUE during the maturity period. The effects of combining CRNF and ONF on NUE were variable and may be related to essential soil fertility and N fertilizer type. The results of our experiments show that a 70% CRNF combined with ONF (N2) outperforms a 20% improvement in NUE. For the improvement of NUE, our results are those of (Ma et al, 2021; Ma et al., 2022a) and Duan et al. (2019).

Our findings support the initial hypothesis, as discussed in the results and discussion sections, a combination of CRNF and ONF has numerous advantages, including 1) can provide a sufficient supply of soil N in the late growth period of winter wheat; 2) can significantly increase the dry matter accumulation of winter wheat from regreening to anthesis stages 3) synchronized the N demand of winter wheat and improved the NUE and grain yield; 4) improved the soil nutrient structure and effectively promote crop yield. Our results indicate that a combination of CRNF and ONF in different soils may be adjusted to meet crop demand for nutrients according to crop type. However, soil type and climate differences among cropping regions must be considered when changing the combination of ONF and CRNF application. Furthermore, special attention should be given to differences in types of available CRNF among crop species. This necessitates the development of predictive models that integrate climate, soil, and crop factors to optimize CRNF application and ensure maximum efficacy. Furthermore, investigating potential synergies between CRNF and other sustainable agricultural practices, such as conservation tillage and integrated pest management, can unlock new opportunities for environmentally conscious and productive farming systems.

## 5 Conclusion

This study examined the combinations of ONF and CRNF to investigate the soil N supply, winter wheat growth and productivity over two consecutive growing seasons. It has been found that increased CRNF application provides adequate soil N for winter wheat throughout its late growth period. When the N application rate is 240 kg ha^-1^, a combination of (CRNF: ONF =70:30), have increased soil N supply after the regreening stage, improved plant populations in the jointing and anthesis stages, and significantly increase the number of spikes, thereby increasing yield. This combination of CRNF with ONF have increased N accumulation during the anthesis stage, the transfer of N to the grain prior to anthesis is significantly increased, as is the grain N content and distribution ratio. It has been concluded that a controlled-release mixed N fertilizer application plan strategy is beneficial for improving grain characteristics is used to achieve high winter wheat yield and efficient N utilization.

## Data Availability

The raw data supporting the conclusions of this article will be made available by the authors, on request.
